# Branchial Cleft Cyst With Coexisting Tuberculous Lymphadenitis in an Elderly Patient: A Diagnostic Challenge

**DOI:** 10.1002/ccr3.73713

**Published:** 2026-10-09

**Authors:** Aakash Khatiwada, Rahul Mahato, Bishesh Kattel, Prajita Baral, Priyanshu Karn, Rekarna Bartaula

**Affiliations:** ^1^ Birat Medical College Teaching Hospital Morang Nepal; ^2^ Department of Otorhinolaryngology Birat Medical College Teaching Hospital Morang Nepal

**Keywords:** branchial cleft cyst, cervical lymphadenitis, neck mass, tuberculosis

## Abstract

Cystic lateral neck masses in elderly patients require careful evaluation as clinical and cytological findings may be misleading. Histopathology remains essential for definitive diagnosis, and the uncommon coexistence of congenital lesions with tuberculous lymphadenitis should be considered in endemic regions.

## 1 Introduction

Branchial cysts are the congenital retention cysts arising from unobliterated remnants of a branchial cleft [1]. The most common branchial cleft anomalies arise from the second cleft. Around 75% of second branchial cleft abnormalities are cysts. Branchial arch anomalies are the second most common congenital lesions of the head and neck in children after thyroglossal duct anomalies [2]. Branchial cyst patients most often come to seek medical attention between the ages of 11 and 30 years [3]. The presentation of branchial cyst in the elderly is rare and the presence of a neck mass in the elderly usually points the diagnosis towards more malicious pathologies like primary lymphoma, metastatic malignant tumors, and granulomatous infections like tuberculosis, which should be ruled out [4]. Tuberculous lymphadenitis is a well‐known cause of cervical lymphadenopathy and may occasionally present as a cystic lesion, mimicking congenital neck masses. The coexistence of a branchial cleft cyst with tuberculous infection is rare and can pose a diagnostic dilemma both clinically and radiologically [5, 6]. Here, we report a case of a branchial cleft cyst in an elderly patient, which on histopathological examination was found to be associated with tuberculous lymphadenitis, highlighting the importance of considering infectious etiologies in the differential diagnosis of cystic neck masses in older individuals.

## 2 Case History/Examination

A 72‐year‐old female presented to the ENT outpatient department with a painless swelling in the right lateral aspect of the neck, located below the angle of the mandible, for 5 years. The swelling was insidious in onset and gradually progressive; however, it showed a rapid increase in size over the preceding 6 months without any identifiable precipitating factors. There was no history of dysphagia, odynophagia, hoarseness of voice, trauma, fever, night sweats, fatigue, or weight loss.

On examination, otological and nasal findings were unremarkable. Neck examination revealed a solitary, oval‐shaped swelling measuring approximately 1.5 × 1.5 cm in the right lateral neck, inferior to the angle of the mandible. The swelling did not move with deglutition or tongue protrusion. The overlying skin appeared normal, with no erythema or visible pulsations. On palpation, the mass was firm, non‐tender, non‐compressible, and non‐reducible, with freely mobile overlying skin. However, lymphadenopathy was not present on neck examination.

## 3 Methods (Differential Diagnosis, Investigations, and Treatment)

Based on clinical findings, differential diagnoses included branchial cleft cyst, cystic lymph node, and cystic metastatic lymphadenopathy.

Laboratory investigation revealed hemoglobin level 14.4 g/dL, C‐reactive protein 0.4 mg/dL, Erythrocyte sedimentation rate (ESR) 25 mm/h. Preoperative blood serology included evaluation of HIV, HBsAg, and Anti HCV, which were negative. The thyroid function test was not done. The patient was advised to do a neck ultrasonography, which revealed a well‐defined cystic lesion measuring approximately 27.3 × 20 mm in the inferior aspect of the right parotid region.

Fine Needle Aspiration Cytology (FNAC) yielded approximately 4 mL of straw‐colored fluid. Cytological examination showed paucicellular smears with mature lymphocytes, occasional neutrophils, and histiocytes in a proteinaceous background, suggestive of a benign cystic lesion. No further investigations were done on FNAC aspirate.

A provisional diagnosis of right branchial cleft cyst with reactive Level II lymphadenopathy was made. The patient subsequently underwent surgical excision of the cyst along with Level IIA lymph node dissection. The excised mass was sent for histopathological examination. The patient's post‐operative recovery was uneventful and she was managed with IV antibiotics, IV analgesics and IV fluids.

The surgical site healed well without evidence of hematoma or nerve deficit and was discharged in stable condition. The histopathological report was not available at the time of discharge.

On the follow up visit, the biopsy report of the excised tissue was available which revealed a branchial cleft cyst with caseating granulomatous inflammation. The excised lymph nodes showed caseating granulomatous lymphadenitis consistent with tuberculosis (Figure 1). Ziehl‐Neelsen staining of the tissue demonstrated the presence of acid‐fast bacilli (Figure 2).

**FIGURE 1:**
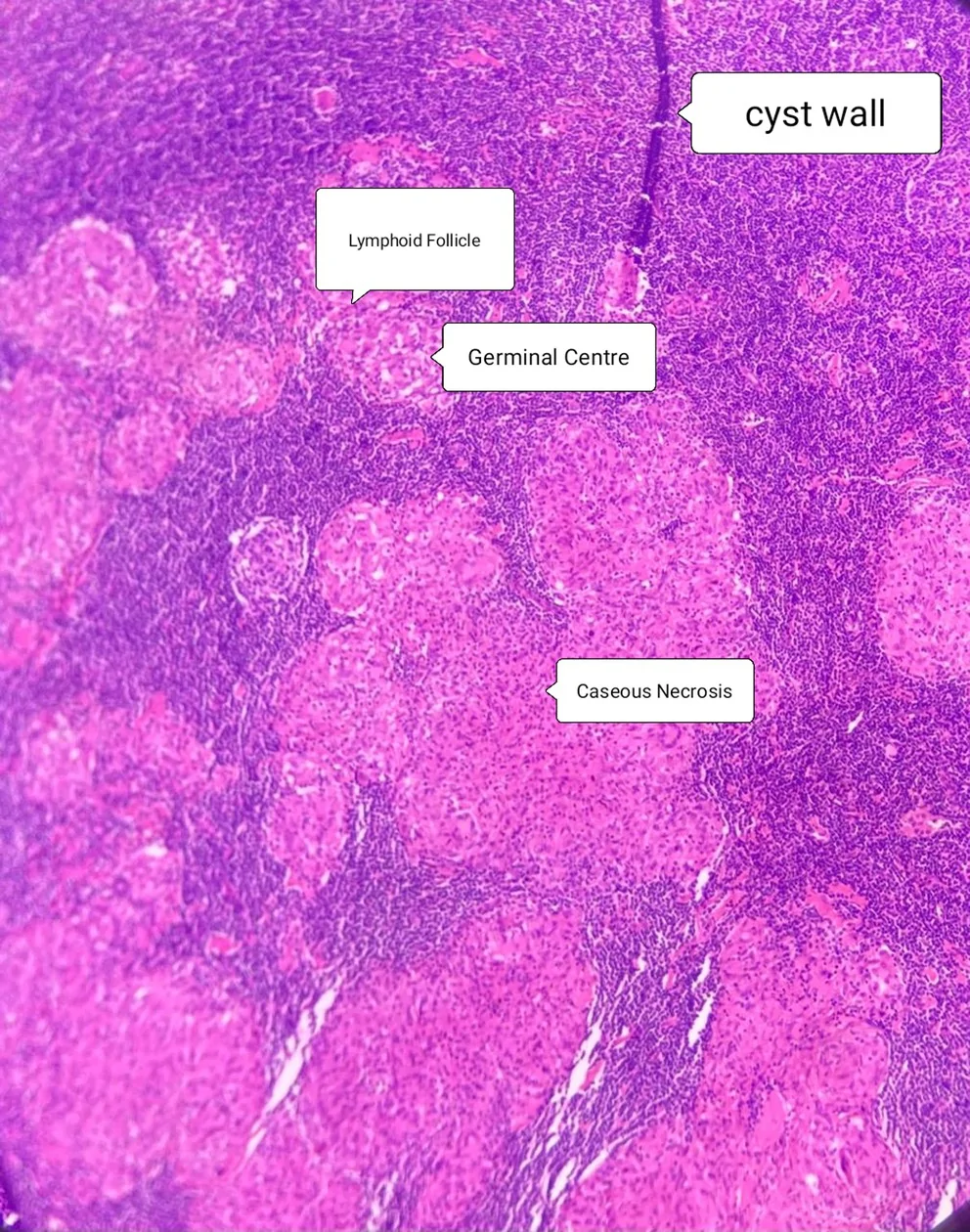
Histopathological slide in 10X showing a cyst wall with lymphoid follicles and germinal centers along with multiple epithelioid granulomas.

**FIGURE 2:**
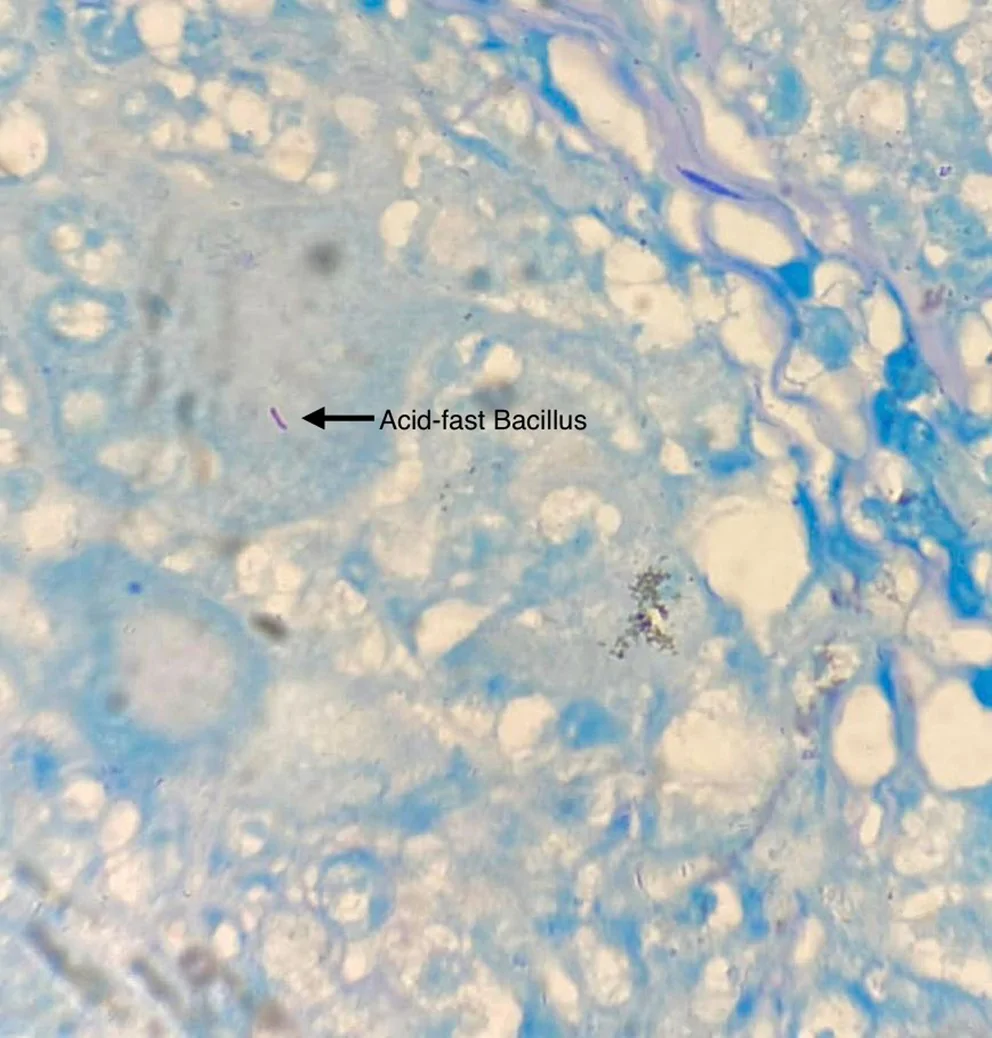
Ziehl‐Neelsen staining showing acid fast bacilli.

## 4 Conclusion and Results (Outcome and Follow‐Up)

On the subsequent follow‐up, granulomatous lymphadenitis was confirmed following which the patient was referred to Directly Observed Treatment Shortcourse (DOTS) centre for further treatment. The patient returned for follow‐up 2 weeks after the initiation of anti‐tubercular medicine. The neck swelling has been absent since the cyst excision, and the surgical site was healing well. She has been taking anti‐tubercular medicine continuously. She has her next follow‐up after one month.

## 5 Discussion

Cystic neck masses in adults and elderly patients represent a diagnostic challenge due to a wide spectrum of differential diagnoses, including congenital lesions, metastatic lymphadenopathy, lymphoma, and infectious etiologies. In this age group, cystic metastatic disease and lymphoma are more commonly encountered than congenital lesions such as branchial cleft cysts [4, 7].

Age is a critical diagnostic factor in the evaluation of lateral neck cysts, as branchial cleft cysts typically present in younger individuals. In elderly patients, a lesion resembling a branchial cleft cyst is atypical and alternative diagnoses should be considered. Current head and neck guidelines recommend that a new neck mass in an adult should be regarded as potentially malignant until proven otherwise, particularly in older patients, where a cystic mass may represent metastatic squamous cell carcinoma rather than a branchial cleft cyst [8]. In our patient, cystic metastatic lymphadenopathy was considered in the initial differential diagnosis. However, despite the advanced age of 72 years, ultrasound and fine needle aspiration cytology (FNAC) favored a benign cystic lesion leading to provisional diagnosis of branchial cleft cyst, emphasizing that congenital lesions should not be excluded solely based on age.

Several studies have evaluated the usefulness of imaging in differentiating tuberculous lymphadenitis from benign and malignant cervical lymphadenopathy. On gray‐scale ultrasonography, features such as strong internal echoes and a thin echogenic rim have been described in tuberculous nodes, while malignant nodes more commonly show loss of the fatty hilum, a rounded shape, and heterogeneous echotexture [9]. Other findings, including nodal matting, intranodal necrosis, and differences in nodal distribution between the posterior triangle and supraclavicular fossa, may also provide useful clues, although there is considerable overlap between tuberculous and metastatic nodes [10]. Doppler investigation provides further information as tuberculous nodes typically show an avascular or displaced hilar vascular pattern, whereas malignant nodes more frequently display peripheral or mixed capsular and hilar vascularity [11]. Sonoelastography has also been done for differentiating benign from malignant lymph nodes. However, its specificity is less in a tuberculosis‐endemic population, particularly when granulomatous inflammation and fibrosis coexist, as these changes can produce a malignant‐appearing pattern [12]. Therefore, despite advances in ultrasonographic assessment, imaging alone does not reliably distinguish tuberculosis from malignancy. In our patient, the imaging findings were similarly unable to establish the underlying diagnosis before surgery. Ultrasonography identified a well‐defined cystic lesion but could not determine its underlying etiology.

FNAC and microbiological tests provide additional evidence but also have important limitations. The reported diagnostic accuracy of FNAC for tuberculous lymphadenitis varies considerably between studies. One Nepalese study reported a sensitivity of approximately 80% and specificity of 100% when histopathology was used as the reference standard, while lower specificity has been reported when mycobacterial culture is used as the reference [13]. This reflects the dependence of cytological diagnosis on sampling adequacy and the presence of caseous necrosis, epithelioid cells, or demonstrable acid‐fast bacilli. GeneXpert MTB/RIF performed on fine‐needle aspirate has therefore been recommended by the World Health Organization as an adjunct to improve detection compared with smear microscopy in high‐burden settings [14]. Clinical scoring systems incorporating factors such as Mantoux reactivity, erythrocyte sedimentation rate, nodal size and number, and constitutional symptoms have also been proposed, particularly in resource‐limited, tuberculosis‐endemic settings [15, 16]. In this case, the absence of typical constitutional features such as fever, night sweats, weight loss, and a discharging sinus would have made tuberculosis less likely on clinical assessment alone.

A similar diagnostic pitfall has also been reported, where a clinically suspected tubercular “scrofula” was ultimately diagnosed as a benign branchial cleft cyst on histopathology [4]. This highlights the considerable overlap between these conditions and the difficulty of establishing a definitive diagnosis based solely on clinical and cytological findings.

In our patient, ultrasound and FNAC findings favored a benign cystic lesion despite the patient's advanced age. However, the excised lymph nodes showed granulomatous lymphadenitis consistent with tuberculosis. Ziehl‐Neelsen staining further demonstrated acid‐fast bacilli, confirming the tuberculous involvement. These findings emphasize the importance of considering tuberculosis even when a cystic lateral neck mass appears clinically and cytologically benign, particularly in tuberculosis‐endemic settings. Also, this case highlights the limitations of ultrasonography and cytology alone and punctuates the need for histopathological evaluation of excised tissue in elderly patients presenting with cystic neck masses.

The distinction between the two conditions changes management. A branchial cleft cyst is generally managed by complete surgical excision [2], whereas tuberculous lymphadenitis is treated with a full course of anti‐tuberculosis chemotherapy, with therapeutic lymph node excision reserved for unusual circumstances [17]. In our patient, anti‐tuberculosis treatment was started on the basis of histological findings and acid‐fast bacilli on Ziehl‐Neelsen staining alone. Further microbiological confirmation (such as Xpert MTB/RIF or mycobacterial culture) and evaluation for pulmonary disease were not pursued because of the patient's financial constraints. This means the mycobacterial species was not confirmed, rifampicin resistance could not be excluded, and undetected pulmonary disease cannot be ruled out. Had the cyst been excised and treated as a branchial cleft cyst without histological examination, the tuberculosis would have gone unrecognized and untreated altogether.

## Author Contributions

**Aakash Khatiwada:** conceptualization, writing – original draft, writing – review and editing. **Rahul Mahato:** conceptualization, investigation, supervision. **Bishesh Kattel:** resources, software, writing – original draft, writing – review and editing. **Prajita Baral:** writing – original draft. **Priyanshu Karn:** resources, software, software. **Rekarna Bartaula:** resources, software.

## Funding

The authors have nothing to report.

## Consent

Informed written consent was obtained from the patient to publish this report in accordance with the journal's patient consent policy.

## Conflicts of Interest

The authors declare no conflicts of interest.

## Data Availability

Data available from corresponding author on reasonable request.
